# Locked-In Syndrome: A Systematic Review of Long-Term Management and Prognosis

**DOI:** 10.7759/cureus.16727

**Published:** 2021-07-29

**Authors:** Taras Halan, Juan Fernando Ortiz, Dinesh Reddy, Abbas Altamimi, Abimbola O Ajibowo, Stephanie P Fabara

**Affiliations:** 1 General Medicine, Ternopil National Medical University, Ternopil, UKR; 2 Neurology, Universidad San Francisco de Quito, Quito, ECU; 3 Neurology, Larkin Community Hospital, Miami, USA; 4 Neurology, Mysore Medical College, Mysore, IND; 5 Emergency Medicine, Amiri Hospital, Kuwait, KWT; 6 Internal Medicine, University Hospitals Cleveland Medical Center, Dallas, USA; 7 General Medicine, Universidad Católica de Santiago de Guayaquil, Guayaquil, ECU

**Keywords:** locked in syndrome, prognosis, long-term care, stroke, long term care

## Abstract

Locked-in syndrome (LIS) is a neurological disorder in which there is damage to the ventral pons and caudal midbrain. An ischemic cause, such as basilar artery occlusion, can often lead to LIS. LIS has three subtypes: classical, partial, and total. There is loss of motion in the four extremities in classical LIS, loss of horizontal gaze, and aphasia. In partial LIS, the patient still has some motor function. Complete LIS has the worst outcome because patients cannot blink or have vertical gaze, thus rendering them incapable of communicating. Most cases of LIS occur due to ischemic infarcts. These patients require a great deal of physical rehabilitation to regain partial motor ability and a means to communicate. While the clinical features and pathophysiology are known, the prognosis and long-term treatment remain unknown.

We conducted a systematic review using the Meta-Analysis Of Observational Studies in Epidemiology (MOOSE) protocol. We use an advanced PubMed strategy using the inclusion criteria of observational studies or clinical trials conducted in the last 20 years, written in English, and conducted on humans. We excluded systematic reviews, literature reviews, metanalysis, and studies that did not meet the outcomes of our objectives.

The prognosis of LIS is not good, and most patients remain locked in, with poor quality of life, especially motor functions. Respiratory failure and depression are big comorbidities. In the acute setting, patients benefit from rapid intervention. The subacute treatment needs to manage aggressively to improve functional scores best. The long-term treatment focus is on the quality of life and managing comorbidities.

## Introduction and background

Stroke is a leading cause of disability, dementia, and death worldwide [1]. Strokes can be divided into hemorrhagic strokes and ischemic strokes [1]. Strokes can lead to brainstem syndromes. There are three subclassifications among brainstem syndromes: midbrain, pontine, and medullary syndromes. Branches of the posterior cerebral artery are involved in midbrain infarcts; the basilar artery and anterior inferior cerebral artery are involved in pons' strokes. Finally, the anterior spinal artery and the posterior inferior cerebellar artery are involved in medullary strokes [2]. In this paper, we will focus on the pontine syndrome, the locked-in syndrome (LIS).

LIS is the most dramatic presentation of a brainstem infarct in the pons [2]. Patients with LIS develop quadriplegia and feel trapped in their bodies. Patients with LIS usually conserve their conscience and can communicate by blinking. LIS syndrome is generally infrequent, but it has been described before [2]. LIS is a complex neurological disorder characterized by quadriplegia, bulbar palsy, and sensory loss [3]. In LIS, there is preserved vertical eye movement, blinking, and level of consciousness [3]. LIS can be caused by occlusion in the mid-basilar artery; it also can be caused by trauma and hemorrhages [4]. Occlusion of the mid-basilar artery results in infarction in the ventral pons but spares the pontine tegmentum [3]. LIS has subtypes: classical, partial, and total LIS.

In classical LIS, there is the characteristic loss of movement in the four extremities and anarthria. In partial LIS, the patient still has some motor function. Complete LIS has the worst outcome because, besides quadriplegia, they cannot blink or have vertical gaze, making them unable to communicate [4]. Patterson and Garbois described 139 patients with LIS; 82 had an infarction in the pons' base. The other patients had different etiologies, such as trauma, central pontine myelinolysis, tumors, encephalitis, neuro-Behcet's disease, multiple sclerosis, and other etiologies of less frequency [5].

Patients have quadriplegia because of the involvement of the corticospinal tracts [6]. The vertical gaze is unaffected because of the nucleus' location in the midbrain's rostral portion [6]. Medial and lateral gaze palsies are common. They can also have diplopia and blurry vision [7] Patients with LIS seem to be unconscious and unaware of their surroundings. However, functional MRI tends to show normal activity [7]. While consciousness is impaired, there has been a report of impaired attention, executive function, and memory [4]. Respiration is often impaired in these patients when the lateral tegmentum is involved [5]. Anarthria is due to paralysis of the facial-glosso-pharyngo-laryngeal muscles and damage of the corticobulbar fibers [7]. Simultaneously, the sensory system is spared most of the time because of the sensory pathways' lateral location. However, the manifestations range from normal to absent [5]. Respiration is generally affected. These patients can present with different respirations patterns like Cheyne-Stokes, apneustic, and ataxic [5]. Table 1 shows the main clinical features of LIS.

**Table 1:** Clinical features of locked-in syndrome

Clinical features		
Quadriplegia	Apneustic respiratory pattern	Preserved consciousness
Anarthria	Emotional liability	Preserved vertical gaze and blinking
Normal or absent sensation	Vertigo	Lateral gaze palsy
Cheyne-Stokes respiratory pattern	Insomnia	Preserved hearing
Ataxic respiratory pattern	Internuclear ophthalmoplegia	Impaired attention and memory (early stages)

While the clinical features, diagnosis, and classification of locked-in syndrome have been established, the treatment and prognosis of this condition remain mainly unknown. We will conduct a systematic review of the prognosis and possible treatment for the rehabilitation of these patients.

## Review

Methods

Search Strategy

We performed a systematic review of the prognosis and treatment of locked-in syndrome. We used the Meta-Analysis Of Observational Studies in Epidemiology (MOOSE) protocol [8-9]. We conducted a search of the last 20 years of articles related to the prognosis and treatment of locked-in syndrome using PubMed as the database for this article.

Eligibility Criteria and Study Selection

The search included any kind of articles except for literature reviews, systematic reviews, and metanalysis. We only included full papers, with humans subjects, and written in the English language. After careful examination, we only included papers with the following characteristics. 1) Population: patients with locked-in syndrome; 2) Intervention: either the quality of life, mortality, or mean years of survival; 3: Comparator: Initial state of locked-in syndrome; 4) Outcome: either prognosis or treatment.

Data Extraction and Analysis

We extracted in each article, the author, country, and year of publication. For the methods, we analyzed the study type, the number of participants, the mean time been diagnosed with locked-in syndrome, and the etiology of the syndrome. Regarding the results, we analyzed the main outcomes of each study related to the prognosis and treatment of this condition, and we wrote the main conclusions of each study.

Bias Assessment

We used the Cochrane Collaboration risk-of-bias tool to analyzed clinical studies, and for observational studies, we used the ROBINS-I tool for observational studies [10-11].

Results

**Figure 1:**
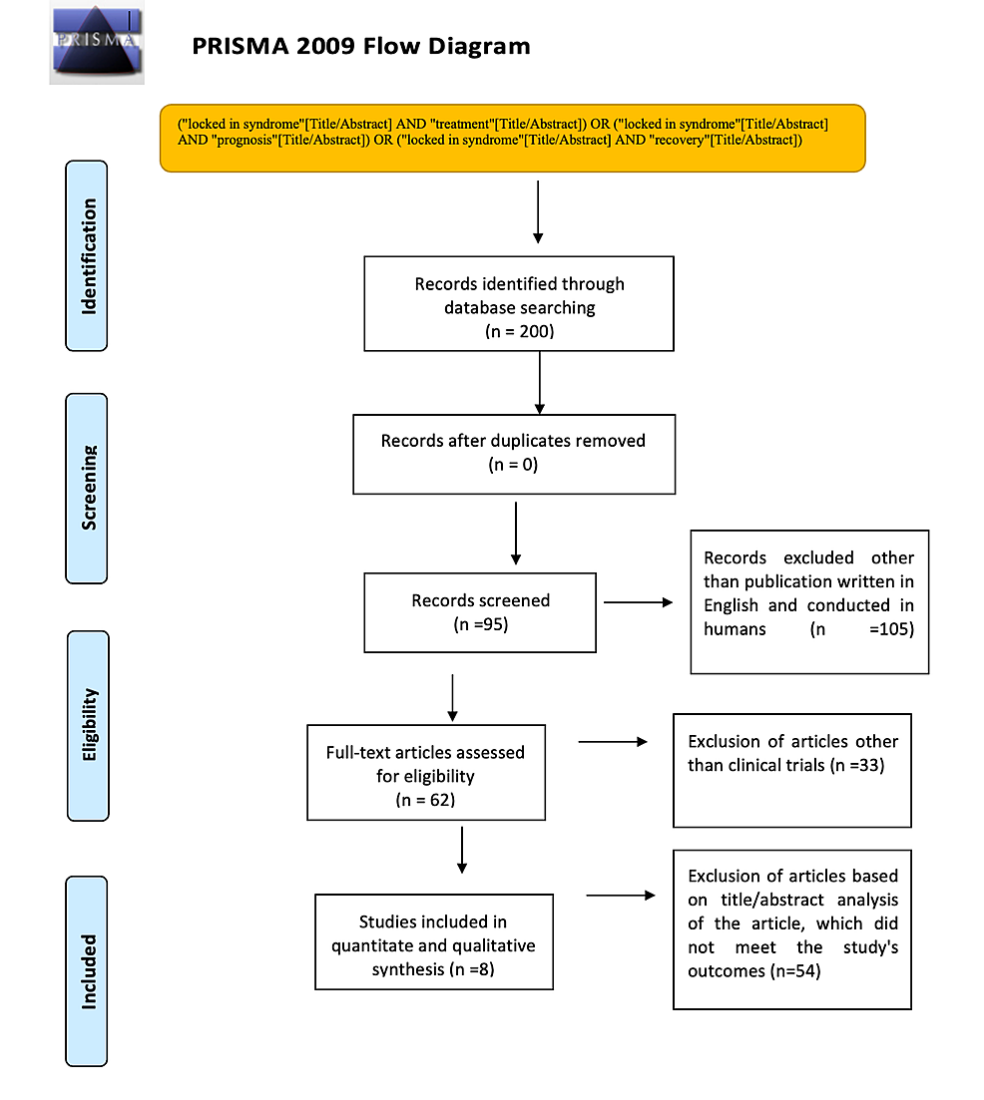
PRISMA flow chart of the systematic review PRISMA: Preferred Reporting Items for Systematic Reviews and Meta-Analyses

Figure 1 details the results of the study in a step-by-step manner.

Study characteristics of the prognosis of LIS [3,11-15].

**Table 2:** Study characteristics of the study

Author, year of publication	Outcome	Country	Participants	Meantime with LIS	Etiology of LIS in patients
Sverling et al, 2019 [12]	Prognosis	Sweden	7/10 were men.; the mean age was 49 years.	Meantime having LIS - 5.9 years.	70% have an ischemic stroke.
Lugo et al, 2015 [13]	Prognosis	France	88 participants (31 females, 57 men). Mean age 52.	Meantime having LIS - 10 +/- 6 years.	Data not reported.
Kohnen et al, 2015 [3]	Prognosis	Holland	187 organizations were asking if they have patients with locked-in syndrome. 12 were reported among all organizations. Two patients have the classic locked-in syndrome. While six have partially locked-in syndrome. Two patients were in a vegetative state, and two patients were excluded.	Meantime having LIS - 6 years.	The majority of patients had an ischemic stroke.
Casanova et al, [14]	Prognosis	Italy	A follow-up of five months to six years of patients with LIS.	Average time of having LIS - 6 years.	Etiology was vascular in 11 cases and traumatic in three cases.
Bruno et al, [15]	Prognosis	Belgium	Five pediatric patients with LIS.	Meantime of having LIS - 14 years.	Stroke in the brainstem ( Four Hemorrhagic and one Ischemic).

Table 3 shows the studies related to the long-term treatment of LIS [16-18].

**Table 3:** Study characteristics regarding the long-term treatment of LIS LIS: locked-in syndrome

Author, year of publication	Outcome	Country	Participants	Intervention
Sacco et al, 2010 [16]	Treatment	Italy	Four patients with locked-in syndrome and opsoclonus-myoclonus syndrome	Training session with psychotherapy and rehabilitation.
Pistola et al, 2014 [17]	Treatment	United States	Four patients with locked-in syndrome and opsoclonus-myoclonus syndrome	Gabapentin 1200 mg.
Hoyer et al, 2014 [18]	Treatment	Norway	A case series of 16 patients with Locked-In syndrome. Only nine patients participate in the study.	Rehabilitation program five times a week that included physical, speech, shallow, and psychological therapy.

Table 4 describes the outcomes of the studies related to prognosis and long-term treatment of LIS [3,12-17].

**Table 4:** Outcomes of the studies related to prognosis and treatment of LIS LIS: locked-in syndrome

Author, year of publication	Problem	Conclusions
Pistola et al, 2010 [17]	opsoclonus-myoclonus syndrome	The patients improve their symptoms and their quality of life. The symptoms resolve quickly and there were long-lasting.
Sacco et al, 2010 [16].	Pathological laughter and crying	After six to eight weeks, patients regain control of their outbursts
Hoyer et al, 2014 [18]	Quality of life.	Physical performance improved in all patients with incomplete LIS. Improvements ranged from walking, practice walking, and better postural control of the head.
Sverling et al, 2019 [12]	Quality of life	There was a decrease of mean type survival of 1.9 years. Seven patients were still alive. Three patients experience a good quality of life. Mortality 30% Three patients experience a good quality of life. All participants report having some improvement.
Lugo et al, 2015 [13]	Quality of life	62% used eye communication with or without assistant technology. 49% use verbal communication, and 73% of patients reported improvement of their eye movements. 92% of patients could establish communication beyond a simple yes or no.
Kohnen et al, 2015 [3]	Quality of life	The study looked at the prevalence and characteristics of LIS in Dutch nursing homes. No treatment/management was examined. The prevalence of LIS in Dutch nursing homes is not entirely indicative of the prevalence of LIS in the entire country due to factors such as patients living outside nursing homes and receiving home care as well as the Dutch practice of end-of-life decision making which included euthanasia or withholding or withdrawing artificial nutrition/hydration.
Casanova et al, [14]	Quality of life	21% of subjects had a motor recovery, 28% had verbal communication, 42% had swallow recovery, 42% communication through devices, effective bladder and bowel control in 35%, good breathing patterns in 50%.
Bruno et al, [15]	Quality of life and Rehabilitation.	35% of pediatric locked-in syndrome patients showed some motor recovery, 26% showed good recovery, 16% remained quadriplegic and aphonic, and 23% died.

Bias Assessment

In Table 5 we use the Robins-1 tool to evaluate the risk in the studies regarding the prognosis and long-term treatment of LIS [3,12-17].

**Table 5:** Bias assessment using the Robins-1 tool

Study	Confounding	Selection bias	Classification of intervention	Deviation from intervention	Missing data	Measurement of the outcome	Selection of reported result
Pistola et al, 2010 [16]	Low	Low risk	Moderate risk	Low risk	Low risk	High risk	Low risk
Sacco et al, 2010 [16]	Low	Low risk	Moderate risk	Low risk	Low risk	High risk	Low risk
Hoyer et al, 2014 [18]	Low	Low risk	Low risk	Low risk	Low risk	High risk	Low risk
Sverling et al, 2019 [12]	Low	Low risk	Low risk	Low risk	Low risk	Low risk	Low risk
Lugo et al, 2015 [13]	Low	Low risk	Low risk	Low risk	Low risk	High risk	Low risk
Kohnen et al, 2015 [3]	Moderate	Moderate risk	Low risk	Low risk	Moderate risk	High risk	Low risk
Casanova et al, [14]	Moderate	Low risk	Low risk	Low risk	Moderate risk	High risk	Low risk
Bruno et al, 2015 [15]	Low	moderate risk	Low risk	Low risk	Moderate risk	High risk	Low risk

Regarding confounding factors, Kohnen et al., including the inability to gather a comprehensive prevalence result due to Netherlands euthanasia or withholding/withdrawing artificial nutrition and hydration in the context of end-of-life decision-making. Additionally, no prevalence comparison was done due to the fact that prevalence data were not available in the literature [3].

In the Casanova et al. study, the evaluation of prognosis and recovery in patients with LIS receiving early intensive rehabilitation care is somewhat confounded given the widely skewed data and the chronological order of the care administered to patients diagnosed with LIS [14].

Discussion

Prognosis

Patients usually do not have a complete recovery. Despite this, the view on prognosis and mortality has drastically improved over the years, yielding positive results [12]. If the patient is medically stabilized and survives the first year where 87% of the deaths occur within the first four months, five-year survival can reach 86% [12]. The survival rate after the 10-year mark of onset is reported to be around 80% [8]. Most of the patients with LIS become dependent on others to carry out activities of daily living due to highly impaired motor function. Studies on quality of life (QoL) have shown that the majority of low scores on QoL stem from motor impairment as opposed to other cognitive/mental reasons. This is not to say that patients with LIS do not suffer from depression or other psychiatric ailments. Mild and moderate depression is more common in patients with LIS than in healthy controls.

Emotional volatility has been documented in such patients and manifests in the form of involuntary laughing or crying, a known problem after injuries to the brainstem [12]. Initial attention span and ability to communicate are affected. However, patients tend to improve with time [8]. Another report of 139 patients demonstrated a mortality rate of 60% in patients with LIS [6]. Most patients continue to be locked in or have significant impairments such as memory loss seen in 18% of the patients [8]. However, cases of partial recovery have been detailed in the literature [18]. A highly complex, as well as significantly prolonged in comparison to the average stroke rehab program, daily inpatient rehabilitation program consisting of occupational, physical, and speech therapy has been shown effective in dramatically increasing the patient’s Freedom Independent Movement (FIM) score as well as provide the ability to complete most activities of daily living (ADLs) with minimal assistance [19]. Pulmonary complications, such as atelectasis and pneumonia, due to aspiration and impaired cough reflex are the leading cause of death in patients with LIS [8].

Patients’ families and their needs have to be taken into account as well. One of the most important needs that have been reported by patients’ relatives was the need for medical information. Of the multitude of topics that related to taking care of the patients, the most important was for LIS’ family members to know that the patient’s needs and wishes were respected by the medical staff. A goal of care form should be signed by the guardian if the patient has does not have one signed previously. Discuss with the family the goal of care if there is one in place by the patient before getting ill, and if not, the person in charge of the guardian should outline the goal of care to the medical team. Receiving accurate medical information is of crucial importance to families of patients with LIS and their quality of life seems to be directly correlated [13].

A Dutch study has shown the very low prevalence of LIS in nursing homes. However, this may stem from two reasons that are somewhat unique to the Netherlands. One reason is that patients with LIS may be receiving home care outside the nursing homes due to the government budget allowance. Another reason is the Dutch end-of-life decision-making, such as euthanasia or withdrawing artificial nutrition/hydration, which decreases the prevalence of patients with LIS [3].

Regarding prognosis in children, blockage of the basilar artery is known to be poor, with a death rate of 25% and severe complications in survivors. However, compared to adult patients, the death rate for vascular etiology is over 75%. Which suggests younger patients have a better prognosis [15]. The prognosis in the same compared to adults was better. Most of the children with LIS show some motor recovery (35%). In 26% of cases, the patient returned to independent living; 16% remained quadriplegic, and 23% died [15].

Long-Term Treatment

Multidisciplinary rehabilitation is the cornerstone treatment of locked-in syndrome. It includes physical, speech, and occupational therapy and assistive devices to get more functional improvement. Also, the treatment could be divided into two categories: immediate and long-term management. In acute settings of LIS, securing the airway and providing sufficient oxygenation is the first step as well as a critical component of acute care [14]. In the acute setting of patients with LIS secondary to ischemic stroke of vertebral or basilar arteries, it is necessary to provide recanalization through intravenous or intraarterial thrombolysis within 48 hours of the onset of symptoms [20].

In terms of subacute and long-term therapies, LIS treatment is aimed to give aggressive supportive measures such as physical, speech, respiratory, swallowing therapy, and as mentioned above, the use of assisted devices. One multidisciplinary rehabilitation study in 2003 aimed at the recovery outcomes of 14 patients. It showed the following data. Three patients gained partial or full independence in daily living activities; six patients were able to swallow completely; four patients recovered verbal communication; six patients were able to use their hands and made finger or head movements, and tracheostomy was removed by six patients [14].

It is imperative that early and intensive multidisciplinary rehabilitation be started within a mean of one month after the onset of the morbid results. According to Casanova's study, this leads to a significantly lower mortality rate, more significant motor recovery, complete swallow recovery, verbal communication, communication through medical devices, and effective bowel and bladder control [14].

The combination of early use, proper goal setting, and multimodal treatments that are frequent and sensory can provide significant improvement in the treatment of patients with LIS. Any and all resources should be utilized to treat the LIS population. Functional electrical stimulation (FES) cycling or a two-channel neuromuscular electrical stimulation (NMES) unit utilizing the principles of neuromuscular re-education paired with repetitive task practice have been found particularly beneficial. Paired with locomotor training, or body-weight supported treadmill training (BWSTT), utilizing the LiteGait harness (LiteGait, Tempe, Arizona) over the treadmill and overground has proven particularly useful for lower extremity therapy. Robot-assisted gait training (RAGT) improves lower limb function, functional ambulation, as well as independence [17].

Upper extremity training utilizing limb robotics, such as the Armeo®Spring program (Hocoma AG, Volketswil, Switzerland), has shown efficacy in increasing accuracy and time of initiation. The repetitive task practice combined with constant visual feedback results in an improvement in self-monitoring, upper limb motor function, automatic motor responses, and sensory feedback patterns. Extremity neuroprosthesis, such as Bioness H200, can be used for functional recovery of grasp in upper extremities, as well as facilitate functional arm use, spasticity reduction, grip strength, and active range of motion [17].

Motor function and postural control improvement in patients with incomplete LIS can be enhanced with the aid of treadmill therapy (TT). TT is a safe and adverse effect-free method that has demonstrated improved physical performance through body weight support assistance in which physical therapists assisted LIS patients with incremental passive movements, which later progressed to more independent body support, which have shown efficacious in postural trunk support, lower extremity mobility, and head control [17].

Regarding LIS complication management, it is essential to monitor respiratory secretions to prevent pneumonia, which is the leading cause of death. These measures include chest physiotherapy and breathing exercises [14].

Bruno et al. conclude that early and intensive rehabilitation can help with verbal communication and functional motor outcomes regarding long-term management in young children. Several studies have found that cognitive abilities are unaffected in the case of a locked-in syndrome in young people caused by a single brainstem lesion. In contrast, individuals with multiple lesions may experience cognitive dysfunction. Treatments that depend on brain plasticity are expected to be more useful in early life as compared to adults [15].

Speech-language and music therapy co-treatment showed improvements in voice output, initiation, as well as coordination. VitalStim using NMES with a speech therapist not only increases airway clearance but it also advances diet status from NPO to puree solids with ice chips. Eye gaze selection can be used for initial communication that later can progress to the Verbally app for functional communication if hand movement is to return [21].

Regarding communication treatment in LIS cases that have been complicated by opsoclonus-myoclonus, gabapentin showed extremely promising results. Opsoclonus-myoclonus is a condition in which the eyes seemingly randomly and involuntarily move rapidly in the horizontal, vertical, and diagonal directions [17]. This makes communications extremely difficult, if not impossible, for patients relying on eye communication. One study noted that the administration of gabapentin resulted in rapid and long-lasting resolution of myoclonus-opsoclonus without any adverse effects by reversing opsoclonus symptoms by acting as a regulator of the saccadic circuit's gain; it might exert its effect either by enhancing pause neurons or by hindering the burst ones [17].

Among many treatment approaches, one particular focus has to be lent to the instance of pathological laughter and crying (PLC). PLC, not having any relation to any mood disorders, is hypothesized to be due to direct damage to the pontine center or an alteration in the pinto-cerebellar pathway linked to emotional behavior to contextual information. Pharmacological treatment with antidepressants has not shown concrete results. However, cognitive behavior may speed up or improve any possibility of spontaneous recovery in six to eight weeks [16].

Overall early intervention and the quality of long-term treatment make a difference in patients with LIS.

## Conclusions

On average, patients with LIS syndrome have a poor prognosis, and most patients remain locked in, regaining a small amount of capacity. QoL scores remain low through the years, especially regarding the motor aspect. Depression is also a long-term complication of patients with LIS. Overall, children with LIS have a better prognosis than adults.

Multidisciplinary rehabilitation is the cornerstone of the rehabilitation of LIS. In the acute setting, rapid intervention is key for long-term prognosis. In the subacute setting, aggressive measures with speech, physical, respiratory, and swallowing need to be undertaking. Long-term therapy includes managing the quality of life and preventing comorbidities. As time progresses, regaining independence is more difficult for LIS patients. Speech therapy, respiratory therapy, visual stimulation, and music therapy are some of the long-term therapies included in the long-term care of the patients.
